# The Need to Anticoagulate Patients With Splenic Vein Thrombosis

**DOI:** 10.7759/cureus.26488

**Published:** 2022-07-01

**Authors:** Clara Benjamin, Maya Bryant, Tri Tran, Rediet T Atalay, Girma M Ayele, Miriam B Michael

**Affiliations:** 1 Internal Medicine, Howard University College of Medicine, Washington DC, USA; 2 Internal Medicine, Howard University Hospital, Washington DC, USA; 3 Internal Medicine, Howard University, Washingon DC, USA; 4 Internal Medicine, University of Maryland, Baltimore, USA

**Keywords:** alcohol, gastrointestinal bleeding, pancreatitis, splenic vein thrombosis, anticoagulation

## Abstract

Splenic vein thrombosis (SVT) is a well-recognized complication of acute and chronic pancreatitis. It is associated with complications of significant gastrointestinal bleeding and high morbidity if the thrombus propagates. There is a need to consider several factors in choosing whether to anticoagulate the patient. We report a case of SVT in a patient with a previous history of pancreatitis who presented with abdominal pain, nausea, and vomiting to the hospital. At the hospital, a CT scan revealed SVT. This case highlights the importance of undergoing further studies regarding anticoagulation for treating SVT in patients at risk for gastrointestinal bleeding.

## Introduction

Splenic vein thrombosis (SVT) mainly occurs due to acute and chronic pancreatitis. It is usually asymptomatic, but patients can present with anemia, hematemesis, melena, and abdominal pain [1]. Unlike other causes of SVT, such as malignancy, the factors associated with pancreatitis-related SVT are caused by transient factors such as local thrombotic inflammatory changes, extrinsic splenic vein compression from pseudocyst, or relatively reduced pancreatic perfusion [2]. Due to the advancement in imaging techniques, such as sensitive CT scans, MRIs, and ultrasound, SVT has become easier to identify [2].

Gastric variceal bleeding is an uncommon complication of SVT. Anatomically, the splenic vein is found posteriorly to the tail of the pancreas, so repeated irritation of the pancreas can result in direct damage to the vein, external compression, or thrombosis [1]. This can lead to gastric varices and an increased risk of bleeding. This can be diagnosed endoscopically.

## Case presentation

A 54-year-old female presented to the hospital with a history of alcohol use disorder, hypertension, gastrointestinal (GI) bleeding due to alcoholic gastritis, type 2 diabetes mellitus, hyperlipidemia, and colon cancer status post-hemicolectomy nine months prior, with a previous admission of acute pancreatitis. She has a history of alcoholic gastritis in the past and was admitted and treated for that.

She presented with sharp, burning epigastric and left upper quadrant (LUQ) pain for two days, accompanied by nausea, vomiting, loose stool, and tea-colored urine. Her pain was persistent, and she continued to have nausea with vomiting but no hemoptysis, hematochezia, melena, or bright red blood per rectum. Physical examination of the abdomen did not show shifting dullness or fluid waves. She had guarding, rigidity, and tenderness to palpation, which was greatest in the LUQ.

Upon admission, she had her laboratory done (Table 1), which was significant for leukocytosis with a white blood cell count measuring 22.59/uL and hemoglobin 16.1 g/dL, which quickly resolved with hydration and supportive therapy. Other pertinent lab findings were noted in the patient’s hepatic panel: Lipase level was significantly elevated, measuring at 2567 units, and amylase at 148 units.

**Table 1:** Laboratory results

Test	Results	Normal
WBC	22,590, high	5000-10,000/uL
Hemoglobin	16.1, high	12-18 g/dL
Lipase	2567, high	0-160 u/L
Amylase	148, high	60-120 units
Total bilirubin	1.3	01.1-1.2 mg/dL
Direct bilirubin	0.4	<0.3 mg/dL
Aspartate aminotransferase	54	15-37 u/L
Alanine aminotransferase	63	30-65 u/L

**Figure 1:**
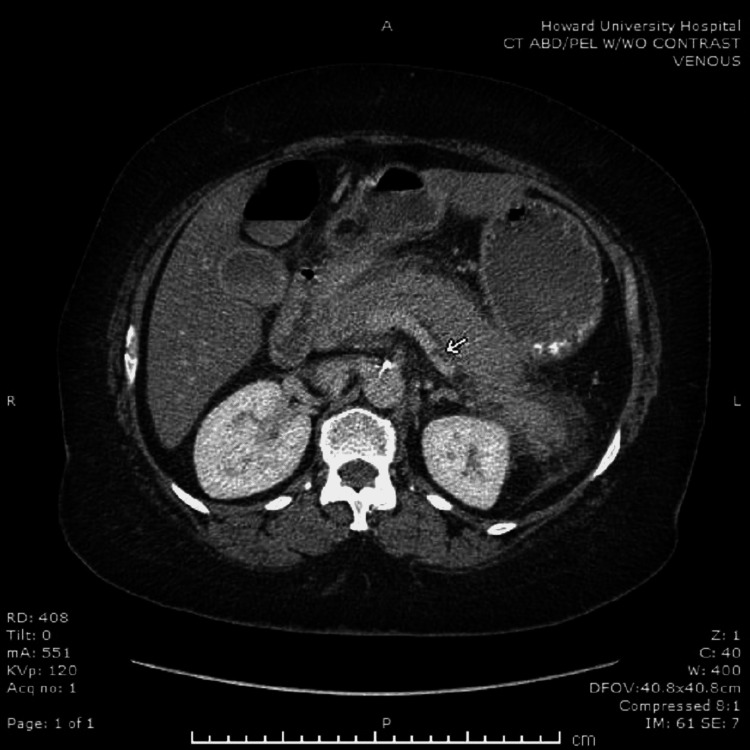
Abdominal CT scan without contrast showing thrombus in the splenic vein (white arrow)

The abdominopelvic CT (Figure 1) conducted on the day of admission revealed SVT, acute pancreatitis, and steatosis. A follow-up CT done after three days demonstrated spontaneous improvement of SVT compared to initial findings without any anticoagulation. During her stay, her symptoms improved, her electrolytes were corrected, and she was counseled on alcohol cessation.

## Discussion

Many different etiologies cause SVT, but most are related to inflammation of the pancreas. Chronic pancreatitis followed by acute pancreatitis and pancreatic neoplasm is the leading cause provoking the process. There are many competing issues in deciding whether or not to anticoagulant patients with SVT. Splenic vein thrombosis creates localized portal hypertension commonly referred to as "sinistral," "left-sided," or "linear" [3]. Collateral blood flow develops through the splenoportal or gastroepiploic systems, and the resulting localized venous hypertension may produce gastric, esophageal, or colonic varices. These varices are sources of significant GI bleeding in 12% of the patients [3]. Since most patients with SVT are asymptomatic and since there is a 30% chance of recanalization, for assessing the coronary vein drainage, patients with a high risk of developing esophageal varices were picked [3]. Routine endoscopy, the gold standard for identifying esophageal varices, should also be considered [4]. Splenomegaly is not always seen in patients with SVT and hence is not a reliable marker of sinistral hypertension [3].

Ascites from extensive splanchnic thrombosis are a further concerning symptom. It appears to be associated with significant morbidity and mortality, so it is recommended to start anticoagulation in this setting or if there is the propagation of thrombosis into the main or intrahepatic portal vein [4].

Based on the results of a large prospective multicenter study, the European Network for Vascular Disorders of the Liver (EN-Vie) recommended the utilization of early anticoagulation in patients with acute portal vein (PV) thrombosis in non-cirrhotic, non-malignant patients. Furthermore, splenic vein (SV) thrombosis associated with ascites was found to have a poor prognosis, suggesting that such patients should receive anticoagulation or thrombolysis [5]. The type of anticoagulation (heparin or vitamin K antagonists) administered does not appear to influence recanalization [5].

In a study out of London, no significant differences were observed in recanalization rates following anticoagulation (p = 0.076). No complications associated with systemic anticoagulation occurred. One patient developed liver failure related to progressive PV thrombosis, and one patient died. In one systemic review, there were a total of 198 patients in the study, of whom 92 (46.5%) received anticoagulation therapy. The rates of recanalization of veins in the treated and non-treated groups were 14% and 11%, respectively, and bleeding complications were 16% and 5%, respectively [6]. In a recent meta-analysis in 2020, a total of 252 patients with acute pancreatitis induced SVT, of whom 112 patients received anticoagulation (AC) and the remaining 140 patients did not receive any AC. Current studies show no difference in outcomes with and without the treatment of AC and even show a slightly increased risk of bleeding [7].

Controlling variceal bleeding coming from the pancreatic bed could be more challenging compared to arterial bleeding, which can be controlled by radiological embolization [6]. The commonly used techniques used to control variceal bleeding such as portosystemic shunts, splanchnic vasoconstrictors, or a tamponade fails to be effective in variceal bleeding from severe acute pancreatitis. Splenectomy happens to be a highly effective treatment for GI related to splenic venous thrombosis [8]. In patients unfit for surgery, embolization could be the sole treatment [8].

## Conclusions

SVT is a common occurrence in the setting of pancreatic inflammation, and it is associated with pancreatic necrosis peripancreatic collections. Recanalization is observed in almost a third of patients, irrespective of whether or not they receive systemic anticoagulation. There is no clear literature written regarding the risks versus benefits of anticoagulation in the treatment of SVT. Multiple factors appear to need consideration such as acuity of the clot as well as the propagation of thrombosis into the main or intrahepatic portal vein and new-onset ascites as indicators to start anticoagulation. These need to be weighed against the real risk of bleeding. The use of diagnostic studies, CT or ultrasound, and possible endoscopic gastro-duodenoscopy (EGD) to look for varices as well as the presence of cirrhosis that increases the risk of significant GI bleeding may help with the decision. The increased rate of morbidity and mortality in cases of both under or optimal treatment with anticoagulation gives a good ground for undergoing randomized controlled studies.
